# *Staphylococcus aureus* nasal carriage is associated with faster symptom resolution following nasal allergen challenge in ragweed-allergic participants: a subset of the Allergic Rhinitis Microbiome Study

**DOI:** 10.1186/s13223-025-00990-3

**Published:** 2025-11-19

**Authors:** Sophia Linton, Lubnaa Hossenbaccus, Abigail Davis, Jenny Thiele, Sarah Garvey, Hannah Botting, Lisa Steacy, Prameet M. Sheth, Anne K. Ellis

**Affiliations:** 1 Division of Allergy & Immunology, Department of Medicine, Queen’s University, Kingston, Canada; 2 Allergy Research Unit, Kingston Health Science Centre – KGH Site, Kingston, ON Canada; 3 Department of Biomedical and Molecular Sciences, Queen’s University, Kingston, Canada; 4 Gastrointestinal Diseases Research Unit, Kingston Health Sciences Centre, Kingston, Canada; 5 Division of Microbiology, Kingston Health Sciences Center, Kingston, ON Canada; 6 Department of Pathology and Molecular Medicine, Queen’s University, Kingston, Canada

**Keywords:** Nasal allergen challenge, Ragweed, *Staphylococcus aureus*, Nasal carriage, Symptoms

## Abstract

In this letter, we report that ragweed-allergic participants with nasal *Staphylococcus aureus* carriage (n = 7) exhibited significantly smaller reductions in Peak Nasal Inspiratory Flow from baseline at 3 h (*P* = 0.013) and 5 h (*P* = 0.008) post–nasal allergen challenge compared to non-carriers (n = 12). There was no significant difference between carriers and non-carriers in the initial response within the first three hours following the challenge (all *P* > 0.05). Carriers also reported significantly lower Total Nasal Symptom Scores (*P* = 0.015) and Total Rhinoconjunctivitis Symptom Scores (*P* = 0.021) at 48 h. These findings suggest that *S. aureus* carriage does not exacerbate allergic responses and may instead be associated with more rapid symptom resolution.

*Staphylococcus aureus*, a Gram-positive bacterium colonizing ~ 30% of individuals, has been implicated in allergic rhinitis (AR) [1]. While some studies suggest higher carriage rates in AR patients [2–7], evidence linking *S. aureus* to symptom severity remains conflicting [4], and its role during controlled nasal allergen challenge (NAC) has not been established.

We previously performed the Allergic Rhinitis Microbiome Study (ARMS), whereby ragweed-allergic participants were challenged to ragweed extract using a NAC outside of the Southeastern Ontario seasonal allergy seasons [8]. Nasal swab samples were collected from all participants at the screening visit to determine methicillin-sensitive *S. aureus* carriage. Using a sterile Transystem™ (COPAN Diagnostics, California, USA) swab set, the anterior nares of one nostril was swabbed and placed in a transport media. Samples were processed for culture and antibiotic susceptibility testing by the Clinical Microbiology Laboratory at Kingston Health Sciences Centre - KGH Site. This study received ethical approval by the Queen’s University Health Sciences and Affiliated Teaching Hospitals Research Ethics Board.

Of 19 ragweed-allergic participants, 7 (38.9%) were *S. aureus* carriers. Self-reported clinical symptom outcomes of mean Total Nasal Symptom Score (TNSS), Total Rhinoconjunctivitis Symptom Score (TRSS), Total Ocular Symptom Score (TOSS), and Peak Nasal Inspiratory Flow (PNIF) were stratified based on *S. aureus* nasal carriage status (Fig. 1). One allergic participant was excluded due to incomplete symptom diary cards. We observed that allergic participants with the presence of *S. aureus* in their nasal microbiome experienced significantly reduced TNSS and TRSS at 48 h post-NAC (TNSS, *P* = 0.015; TRSS, *P* = 0.021) compared to those without. TOSS was not significantly different between these participant groups at any time-point (*P* > 0.05*)*. To account for differing baseline scores between participants, mean PNIF scores were normalized as mean percent PNIF fall from baseline. Allergic participants who were carriers of nasal *S. aureus* experienced significantly reduced percent PNIF fall at the 3 h (*P* = 0.013) and 5 h (*P* = 0.008) time-points compared to non-carrier participants.

**Fig. 1:**
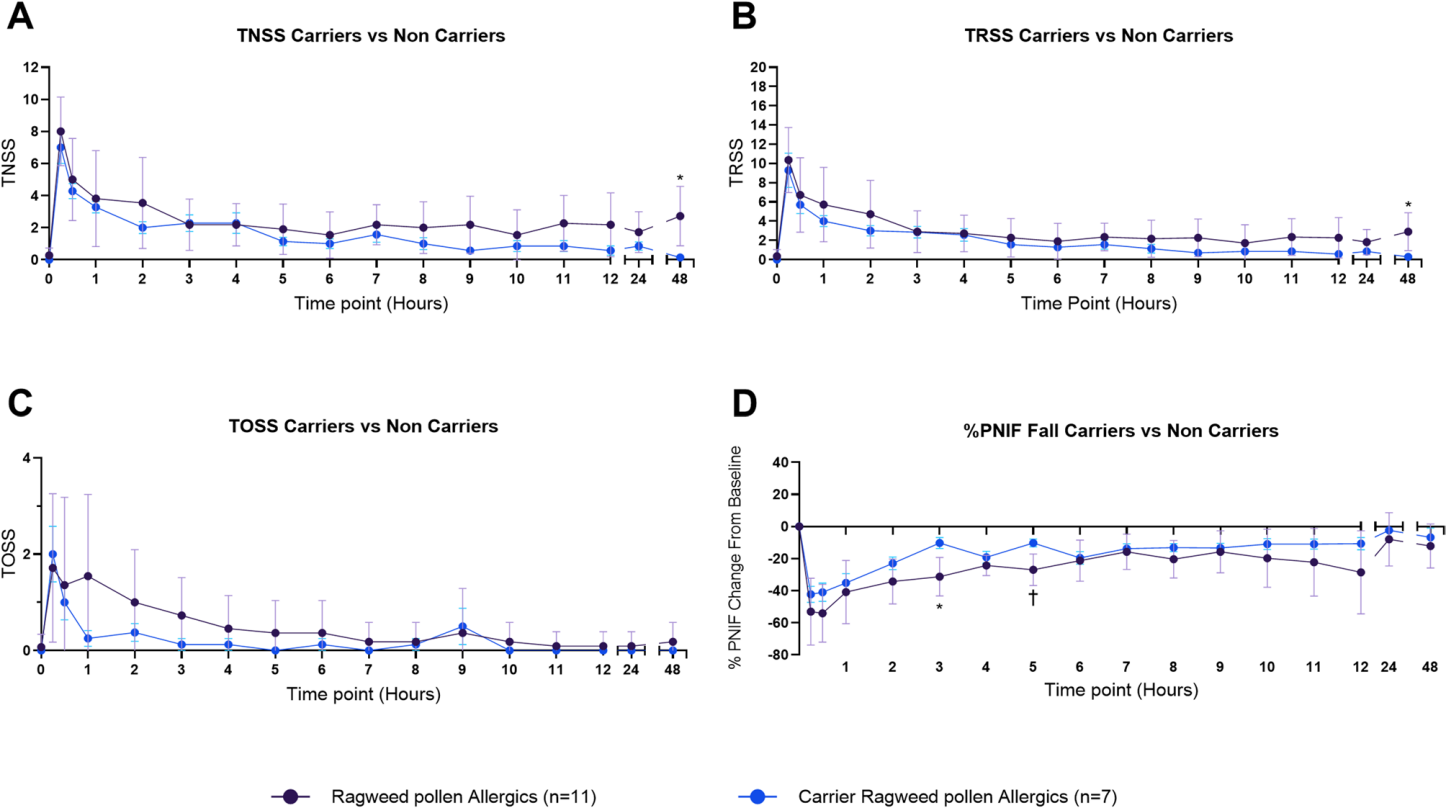
Clinical outcomes during nasal allergen challenge in ragweed-allergic participants with (carriers) and without (non-carriers) nasal *Staphylococcus aureus*. Mean Total Nasal Symptom Score (TNSS; **A**), Total Rhinoconjunctivitis Symptom Score (TRSS; **B**), Total Ocular Symptom Score (TOSS; **C**), and normalized percent Peak Nasal Inspiratory Flow (PNIF) fall (**D**) are shown. Compared to carriers, non-carriers had significantly higher TNSS (*P* = 0.015) and TRSS (*P* = 0.021) at 48 h post-challenge, as well as greater PNIF fall at 3 h (*P* = 0.013) and 5 h (*P* = 0.008). No significant differences were observed in TOSS. Statistical significance was determined using two-way ANOVA with Šídák’s multiple comparisons test. *P ≤ 0.05; †*P* ≤ 0.01. Error bars represent standard deviation

There is disagreement on whether *S. aureus* impacts the severity of symptoms of AR and other atopic conditions, but considering that 30% of individuals with AR are carriers, it is important that the effects of *S. aureus* are understood [9]. As the first study to use an optimized and validated model of AR in ragweed-allergic participants, we were unable to replicate the findings from the group of Shiomori et al. [4] Although *S. aureus* carriage did not reduce early-phase symptoms, carriers experienced faster symptom resolution, suggesting a potential modulatory role in allergic inflammation. The mean TNSS difference at 48 h between carriers and non-carriers (2.58 points) falls within the range of minimally clinically important differences (1–3.6 points), suggesting a potentially clinically meaningful effect [10, 11]. PNIF measurements showed transient differences during the late-phase response; however, given the effort-dependent nature of PNIF and the small sample size, these changes should be interpreted cautiously. Larger, longitudinal studies are needed to confirm these findings and to explore mechanisms, including the contribution of Staphylococcal toxins to AR and other atopic conditions.

## Data Availability

The datasets generated and/or analysed during the current study are not publicly available as they are proprietary to Kingston Allergy Research but are available from the corresponding author on reasonable request.

## Abbreviations

AR: Allergic rhinitis
ARMS: Allergic rhinitis microbiome study
PNIF: Peak nasal inspiratory flow
NAC: Nasal allergen challenge
TNSS: Total nasal symptom score
TOSS: Total ocular symptom score
TRSS: Total rhinoconjunctivitis symptom score
